# Long-term treatment with streptozocin/5-fluorouracil chemotherapy in patients with metastatic pancreatic neuroendocrine tumors: Case series

**DOI:** 10.1097/MD.0000000000028610

**Published:** 2022-01-28

**Authors:** Christian Müller, Michael C. Kreissl, Silke Klose, Andreas Krause, Verena Keitel, Marino Venerito

**Affiliations:** a Otto-von-Guericke University Hospital, Department of Gastroenterology, Hepatology and Infectious Diseases, Leipziger Str. 44, Magdeburg, Germany; b Otto-von-Guericke University Hospital, Division of Nuclear Medicine, Department of Radiology and Nuclear Medicine, Leipziger Str. 44, Magdeburg, Germany; c Otto-von-Guericke University Hospital, Department of Nephrology and Hypertension, Diabetes and Endocrinology, Leipziger Str. 44, Magdeburg, Germany; d Esteve Pharmaceuticals GmbH, Department of Medicine and Science, Hohenzollerndamm 150-151, Berlin, Germany.

**Keywords:** 5-FU, chemotherapy, neuroendocrine tumor of the pancreas, streptozocin

## Abstract

**Rationale:**

Pancreatic neuroendocrine tumors (pNETs) are rare entities representing 1% to 3% of all malignant pancreatic neoplasms. Current guidelines recommend a combination of streptozocin (STZ) and 5-fluorouracil (5-FU) for patients with metastatic well-differentiated pNETs requiring systemic therapy. The highest median progression-free survival rate reported in previous studies for this combination was 23 months (95% confidence interval 14.5–31.5). However, it remains unclear for how long this regimen can be safely administered.

**Patient concerns:**

We report about 3 therapy-naïve patients with metastatic G2 (Ki67 10%–15%) pNETs treated with STZ/5-FU, that achieved sustained disease control for longer than 36 months.

**Diagnosis:**

Metastatic, well-differentiated G2 pNETs

**Interventions:**

Systemic chemotherapy with STZ/5-FU was administered until the disease progressed. In 1 case showing a mixed response, selected metastases of increasing size were additionally treated with surgery and brachytherapy.

**Outcomes:**

In our 3 patients with metastatic G2 pNETs, STZ/5-FU induced long-term disease control over 44, 42, and 95 months, respectively. No side effects that led to treatment discontinuation were observed.

**Lessons:**

In patients with metastatic G2 pNETs achieving disease control, STZ/5-FU can be safely administered.

## 1 Introduction

Pancreatic neuroendocrine tumors (pNETs) are rare malignancies with an overall incidence of <1/100.000,^[1]^ and they constitute only approximately 3% of all pancreatic neoplasms.^[2]^ They account for a stable proportion of approximately 7% of all neuroendocrine tumors (NETs) over the last few decades.^[3]^ pNETs commonly occur between the sixth and eighth decades of life,^[4–6]^ and males are affected slightly more often than females.^[4,5]^ Patients with genetic predispositions, such as multiple endocrine neoplasia type 1 and von Hippel-Lindau syndrome, have a significantly increased risk of developing pNETs and show an earlier onset of the disease.^[7–9]^ In most cases, pNETs have an indolent clinical course and are first diagnosed when the disease is locally advanced or metastatic.^[10–12]^ pNETs are characterized by a very heterogeneous biology and can present either with symptomatic hormone production (gastrinoma, insulinoma, glucagonoma, VIPoma),^[13–16]^ or with no hormone production.^[17]^ Prognostic factors for patients with pNETs include age, performance status, tumor stage, serum chromogranin A levels, and the tumor proliferation marker Ki-67, whereas predictive markers are still lacking.^[18–23]^ Currently, surgical resection is the only curative therapeutic approach.^[24]^ For patients with unresectable disease, numerous therapies are available, including interferon, somatostatin analogs, chemotherapy with streptozocin (STZ) plus 5-fluorouracil (5-FU) or temozolomide plus capecitabine, targeted therapies with everolimus or sunitinib, and peptide receptor radionuclide therapy.^[24–30]^ According to current guidelines, systemic chemotherapy with STZ/5-FU is the standard first-line therapy to induce disease control in patients with advanced pNETs not amenable to resection.^[31–33]^ This recommendation is based on the trials of Moertel et al,^[34,35]^ who achieved remission in 43% to 63%, as well as an overall survival of 26 to 42 months in patients with metastatic or locally advanced pNETs treated with STZ/5-FU or STZ/doxorubicin. However, it is unclear how long these regimens can be safely administered and how long remission can be maintained. Here, we present 3 patients with pNETs in whom STZ/5-FU induced long-term disease control and was safely administered over the years.

## 2 Case reports

A detailed summary of patient characteristics, tumor-specific data, and clinical course data is presented in Table 1.

**Table 1:** Summary of patient characteristics, tumor specific data, and treatment data of the 3 presented cases.

	Case 1	Case 2	Case 3
Patient characteristics:			
Gender	Male	Male	Female
Year of diagnosis	2014	2016	2009
Age at diagnosis	77	52	53
ECOG performance status	0	0	0
Genetic predisposition	No	MEN1	No
Tumor specific data:			
Tumor grading	G2	G2	G2
Ki-67 index	10%	15%	10%
Hormone secretion	No	No	No
Metastatic site(s)	Liver, adrenal gland	Liver, bone	Liver, lymphatic, peritoneal
Somatostatin receptor (SSR) status	n.i.	Positive	Positive
Chromogranin A level at diagnosis (μg/L)	1109	171	337^∗^
Treatment characteristics			
Prior treatment	None	None	None
Number of streptozocin/5-fluorouracil cycles	41	56	86
Treatment period (mos)	44	42	95
Total amount of streptozocin/ (in g)	72.5	57.6	134.0
Best tumor response	PR	PR	PR
Time to best tumor response (mos)	5	8	3
PFS (in mos)	59	41	37/33^†^
OS (in mos)	59	55	136

ECOG = Eastern Cooperative Oncology Group, n.i. = not investigated, OS = overall survival, PFS = progression-free survival, PR = partial response.

∗ Chromogranin A at relapse.

† First PFS after complete resection/second PFS after induction of systemic chemotherapy.

### 2.1 Case 1

**Figure 1:**
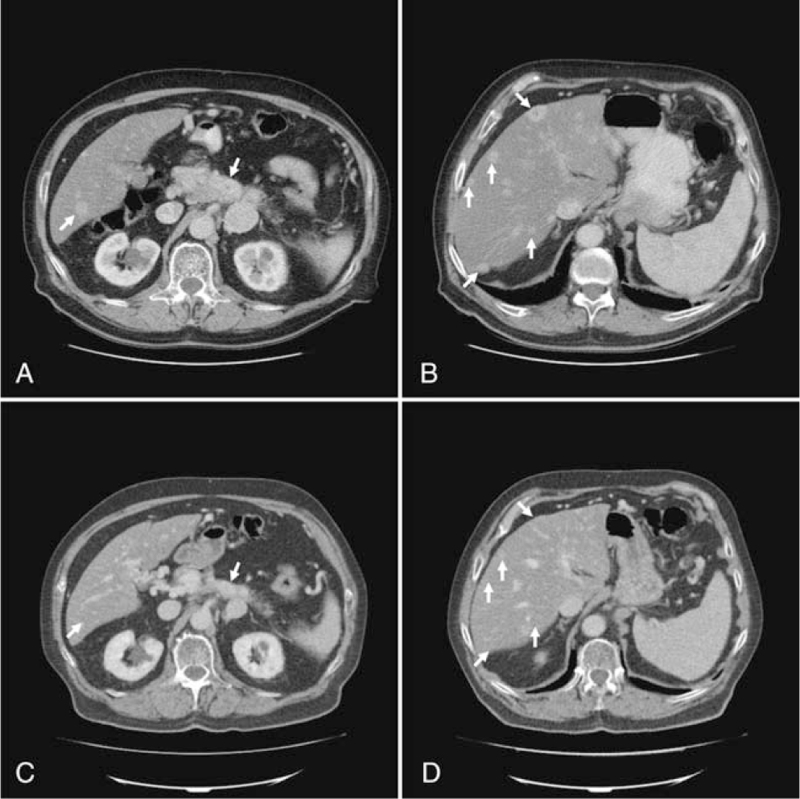
Initial and follow-up imaging of Case 1. (A) The initial contrast-enhanced CT of the abdomen detected a hyperdense lesion within the liver and the pancreatic corpus (white arrow). (B) The initial contrast-enhanced CT imaging demonstrated multiple hyperdense lesions in both liver lobes (white arrows). (C) The follow-up CT showed partial response of the initial hyperdense lesion within the pancreatic corpus after induction of systemic chemotherapy according STZ/5-FU (white arrow). (D) The follow-up CT scan confirmed a partial remission of the known hyperdense hepatic lesions after induction of systemic chemotherapy according STZ/5-FU (white arrows). 5-FU = 5-fluorouracil, CT = computed tomography, STZ = streptozocin.

In August 2014, a 77-year-old Caucasian man presented to our department for diagnostic workup of liver and pancreatic lesions. In 2010, he was diagnosed with prostate cancer (stage 2b, Gleason 3+4 = 7a), which was treated with curative radiation to the prostate and seminal vesicles. During restaging examinations by computed tomography (CT), new lesions within the liver and pancreatic corpus were detected (Fig. 1A and B). Histological examination of the liver biopsy specimen revealed a well-differentiated NET, G2, with a Ki-67 index of 10%. Endoscopic examination excluded a primary tumor outside the pancreas. No pulmonary metastases were observed on the CT of the thorax. In summary, we diagnosed the patient with a hepatic metastatic pNET. The patient received systemic chemotherapy using the STZ/5-FU regimen described by Moertel et al^[34]^ (STZ 500 mg/m^2^ and 5-FU 400 mg/m^2^, days 1–5, qd 43) starting in September 2014. After 4 cycles of chemotherapy, the patient achieved partial remission (Fig. 1 C and D). Overall, he tolerated the therapy well during the first 18 cycles (September 2014 to September 2016), without relevant clinical or laboratory side effects. However, after the 18th cycle, he developed urosepsis with acute renal failure. Blood cultures showed evidence of S*taphylococcus capitis*. After recovery from urosepsis, the glomerular filtration rate was slightly impaired; thus, systemic therapy was restarted without dose alterations. After 3 additional cycles (April 2017) with persistent remission, we switched to the Uppsala regimen (STZ 1000 mg/m^2^ and 5-FU 400 mg/m^2^, d1, qd 22). The patient tolerated this regimen well without relevant clinical side effects or stable retention values for an additional 20 cycles. In total, he received 41 cycles of STZ/5-FU-based therapy over a period of 44 months, with sustained remission. In June 2018, an increase in glomerular filtration rate was observed, and chemotherapy was stopped. The chronic renal failure was the consequence of an obstructive uropathy linked to the presence of a phimosis, a prostate hypertrophy and the previous radiotherapy. The additional etiological contribution of previously administered STZ-based chemotherapy to chronic kidney failure cannot be excluded. After further deterioration of the glomerular filtration rate despite medical and urological therapy, hemodialysis was initiated. The patient died shortly thereafter in August 2018 due to dialysis catheter-associated sepsis.

### 2.2 Case 2

**Figure 2:**
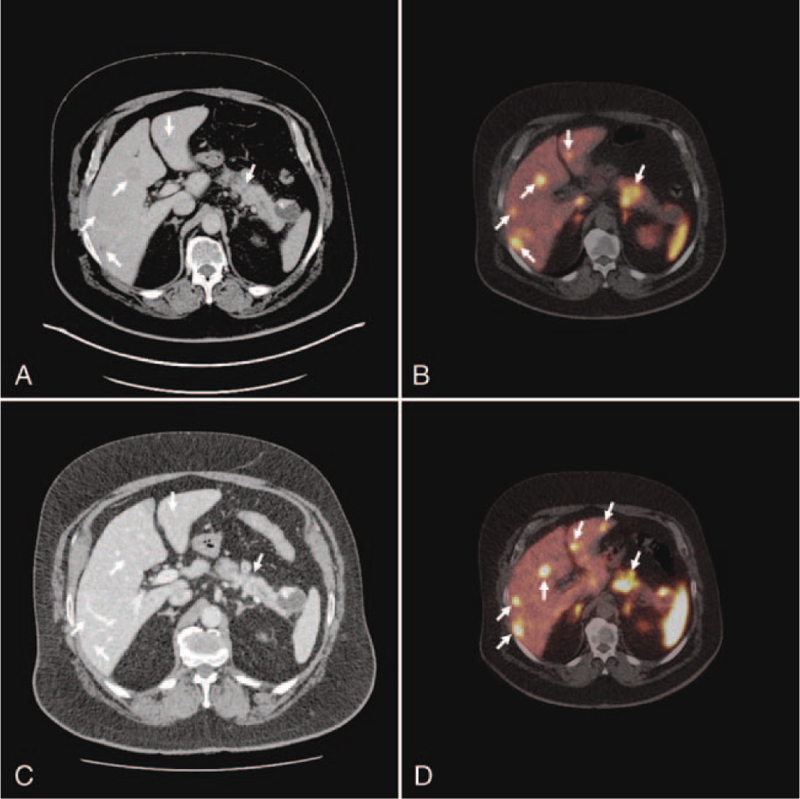
Initial and follow-up imaging of Case 2. (A) The initial CT detected a discrete hyperdense lesion in the pancreatic corpus/tail and hyperperfused areas in both liver lobes (white arrows). (B) The initial Ga-68-DOTANOC PET/CT revealed a large SSR-positive lesion within the pancreatic corpus/tail and multiple SSR-positive lesions within both lobes of the liver (white arrows). (C) The follow-up CT imaging demonstrated partial remission of the hyperdense lesion within the pancreatic corpus/tail and remission of the hepatic metastasis (white arrows). (D) The follow-up Ga-68-DOTANOC PET/CT showed progressive SSR-positive hepatic metastasis in both liver lobes. CT = computed tomography, PET/CT = positron emission tomography/computer tomography, SSR = somatostatin receptor.

A 52-year-old Caucasian man presented to our department in September 2016 with further histological clarification of a liver lesion. He was diagnosed with multiple endocrine neoplasia type 1 (MEN 1), which was responsible for multiple surgeries for parathyroid adenomas in 1990, 1992, and 2004. Because of unclear hepatic lesions detected on a CT scan of the abdomen, we performed a Ga68 DOTANOC positron emission tomography/computer tomography (PET/CT), demonstrating multifocal pathological uptake in the pancreatic tail, both liver lobes, as well as in the thoracic and lumbar spine and the os ilium (Fig. 2A and B). An ultrasound-guided liver biopsy was performed. Histological examination revealed a well-differentiated NET (G2) with a Ki-67 index of 15%. The patient received systemic chemotherapy according to the STZ/5-FU protocol (STZ 400 mg/m^2^ and 5-FU 300 mg/m^2^, days 1–3, qd 22), and achieved stable disease. After 8 cycles (November 2016 to April 2017), systemic therapy was switched to the Uppsala regimen starting in May 2017. Three months later, in August 2017, Ga-68-DOTANOC PET/CT revealed partial remission (Fig. 2C). STZ/5-FU according to Uppsala was continued over a period of 42 months (56 cycles) until disease progression was assessed by Ga-68-DOTANOC PET/CT in May 2020 (Fig. 2D). STZ/5-FU therapy was well tolerated, and no therapy-limiting clinical or laboratory adverse events occurred. Further therapies included everolimus 10 mg daily and peptide radio-receptor therapy, which was started in January 2021.

### 2.3 Case 3

**Figure 3:**
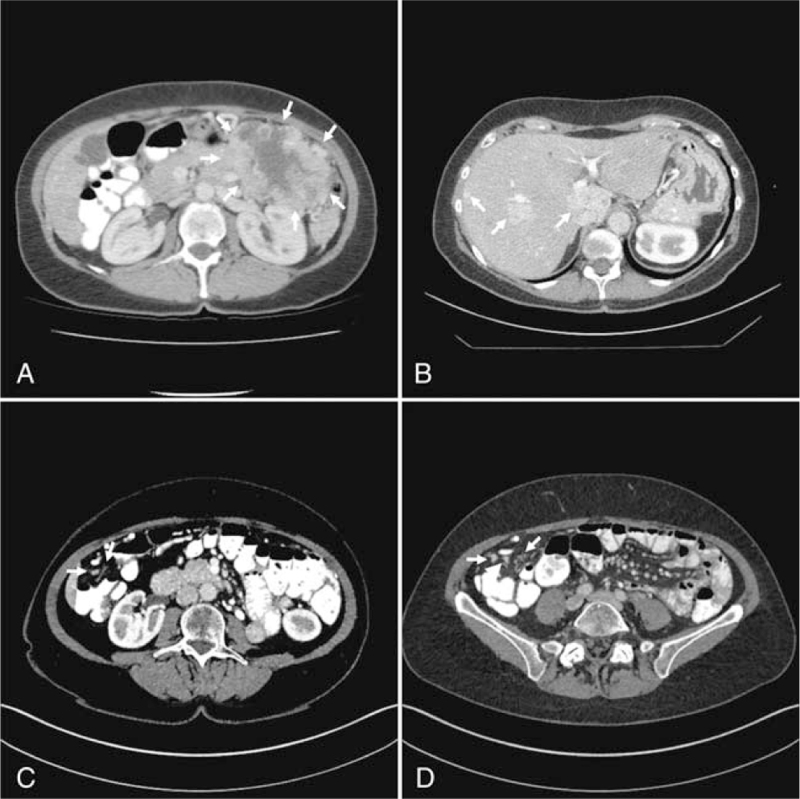
Initial and follow-up imaging of Case 3. (A) The initial contrast-enhanced CT of the abdomen revealed a large inhomogenous tumor bulk in the left upper abdomen affecting the left colonic flexure (white arrows). (B) The follow-up CT showed progressive disease with new liver metastases of the pNET in both liver lobes. (C) The follow-up CT of the abdomen demonstrated progressive peritoneal tumor manifestations in the upper right quadrant (white arrows). (D) The follow-up CT of the abdomen revealed progressive peritoneal tumor manifestations in the lower right quadrant. CT = computed tomography, pNET = pancreatic neuroendocrine tumor.

Our third case involved a 56-year-old woman who presented to our department for the initiation of systemic chemotherapy. In October 2009, the patient presented with non-specific upper abdominal complaints. Abdominal CT revealed an inhomogeneous mass in the pancreatic tail with infiltration of the spleen and transverse colon (Fig. 3A). Ultrasound-guided biopsy of the mass revealed a NET. The initial therapy recommendation was resection of the primary tumor in the absence of pulmonary or hepatic metastases in November 2009, a pancreatic left resection, splenectomy, left-sided adrenalectomy, and resection of the left colonic flexure (histopathological results: well-differentiated NET, G2, Ki-67 index of 10%). After 3 years, in December 2012, new lesions appeared in both lobes of the liver as well as in the right paracolic area (Fig. 3B). However, no radionuclide accumulation was detected on somatostatin receptor (SSR) scintigraphy. Based on these results, she received systemic chemotherapy with the STZ/5-FU protocol according to Moertel et al^[34]^ (STZ 500 mg/m^2^ and 5-FU 400 mg/m^2^ d1–5, qd 43). Partial remission was achieved after 2 cycles. STZ/5-FU was further administered, and the disease control lasted until September 2015. During this period, chemotherapy was well tolerated, no dose reduction or therapy delay was necessary, and no relevant clinical or laboratory side effects were observed. After 22 cycles (January 2013 to August 2015), new lesions in the ileocecal area and ascending colon were observed on a CT scan of the abdomen in September 2015 (Fig. 3C and D), whereas liver metastases were still in remission. These lesions showed additional SSR positivity in the Ga-68-DOTANOC PET/CT performed in October 2015. She underwent debulking surgery with resection of the progressive lesions (omentum majus and uterine tumor). In the resected specimen, a moderately differentiated NET was confirmed, with a Ki-67 index of 20%, which was higher than that of the initial histology. After reconvalescence, systemic therapy with STZ/5-FU was resumed, first according to the Moertel protocol (January 2016 to February 2017), followed by the Uppsala regimen, again with good tolerability and sustained remission for a further 31 months. In September 2018, Ga-68-DOTANOC PET/CT showed a mixed response, with an increase in the size of 2 SSR-positive hepatic metastases in segments I and V/VI. After discussion of possible therapeutic options for the patient, the progressive hepatic lesions were treated with CT-guided interstitial brachytherapy with an Iridium-192 source in November 2018, whereas STZ/5-FU chemotherapy according to the Uppsala regimen was further administered until January 2020. During this period, only an SSR-positive lesion in the pancreatic head increased in size, whereas the remaining NET manifestations were in remission. Therefore, a pancreatic head resection was performed in April 2020. Histopathological examination revealed a poorly differentiated NET, with a Ki-67 index of 35%. After recovery, chemotherapy with STZ/5-FU was resumed, with sustained remission. To date, the patient has received 86 cycles of STZ/5-FU over a period of 95 months.

## 3 Discussion

Herein, we report 3 therapy-naïve patients with metastatic, hormone-inactive pNETs who achieved sustained remission of all or a proportion of tumor manifestations under treatment with STZ/5-FU. All patients were initially treated according to the Moertel protocol and were switched on to the more patient-friendly Uppsala protocol.^[32]^ In 3 cases, partial remission was achieved after 3, 5, and 8 months. Our patients received a median of 56 cycles over a median treatment period of 44 months, with a median progression-free survival of 44.3 months and a median overall survival (mOS) of 59 months.

A similar mOS (54.8 months) was reported by Dilz et al^[36]^ in a German retrospective study of 96 pNET patients who were treated according to the Moertel regimen. However, specific data on the subgroups of patients with pNET with different grades (G1, G2, and G3) are missing in the publication by Dilz et al, which prevented us from making a direct comparison with our results.

Similar to our data, in a retrospective analysis by Antonodimitrakis et al^[37]^ including 133 patients with pNETs of different grades who received STZ/5-FU according to the Uppsala regimen, 14% of the patients received chemotherapy for at least 60 months and 4% for as long as 120 months. In particular, in this study, the authors increased the interval between STZ/5-FU courses in responding patients to allow long-term treatment. A similar strategy was reported in a German study on patients with pNETs of different grades by Schrader et al,^[38]^ where an extended cycle length (3 months) was administered as maintenance therapy after achieving disease control, which was associated with an mOS as long as 69 months. Whether patients achieving disease control with STZ/5-FU may have a higher benefit in terms of survival, safety, and quality of life by continuing the therapy (as in our patients), by increasing the interval between STZ/5-FU courses, as in the above-mentioned studies, or by stopping the therapy is not known. Furthermore, we switched the regimen from induction therapy to the more patient-friendly Uppsala protocol after achieving remission, which may have had a beneficial effect on the quality of life of our patients.

In line with the aforementioned retrospective analyses, long-term STZ/5-FU treatment was safe.^[36–38]^ Indeed, grade 3/4 toxicities leading to dose reduction or discontinuation are rare in patients treated with STZ/5-FU, even over the years. The most common adverse events observed in patients receiving STZ/5-FU were fatigue, diarrhea, stomatitis, nausea, thrombocytopenia, leukopenia, and impaired renal function.^[35–37,39,40]^ The slightly decreased glomerular filtration rate described in case 1 was observed after a uroseptic event and was therefore judged to be unrelated to treatment.

In all 3 cases, we did not observe treatment-related toxicities that led to dose adjustment, an increase in the interval between STZ/5-FU courses, or treatment discontinuation.

## 4 Conclusion

In the case series presented here, we summarized 3 therapy-naïve patients with metastatic G2 pNETs who received long-term treatment with STZ/5-FU for >3 years. Long-term STZ/5-FU therapy is feasible and safe for therapy-naïve patients with metastatic G2 pNETs. Thus, for patients achieving disease control, long-term STZ/5-FU therapy may represent a valuable therapeutic option to achieve long-lasting remission with good tolerability. Prospective trials may further define the optimal maintenance strategy for this selected population.

## Author contributions

CM developed the original idea, drafted the manuscript and tables, and selected figures for the reported cases.

MCK, SK, AK, VK, and MV provided critical feedback, helped shape the manuscript, and were responsible for the important intellectual content.

MV conceived the original idea and supervised the project.

All authors issued final approval for the version to be submitted.

**Conceptualization:** Marino Venerito.

**Data curation:** Christian Mueller.

**Formal analysis:** Christian Mueller.

**Supervision:** Marino Venerito.

**Validation:** Michael Christoph Kreissl, Silke Klose, Andreas Krause, Verena Keitel, Marino Venerito.

**Writing – original draft:** Christian Mueller.
